# Characterization of a chitinase from *Trichinella spiralis* and its immunomodulatory effects on allergic airway inflammation in mice

**DOI:** 10.1186/s13071-024-06656-0

**Published:** 2025-01-13

**Authors:** Jia Xu, Ye Yao, Qisheng Zhuang, Zixuan Li, Min Zhang, Shouan Wang, Hongxin Hu, Jianbin Ye

**Affiliations:** 1 School of Basic Medicine Science, Fujian Province, Putian University, Key Laboratory of Translational Tumor Medicine in , Putian City, 351100 Fujian Province China; 2 School of Pharmacy, Fujian Medical University, Fuzhou City, 350004 Fujian Province China; 3 School of Pharmacy, Putian University, Putian City, 351100 Fujian Province China; 4 The Affiliated Hospital of Putian University, Putian City, 351100 Fujian Province China

**Keywords:** Chitinase, *Trichinella spiralis*, Asthma, Allergy

## Abstract

**Background:**

A fundamental tenet of the hygiene theory is the inverse association between helminth infections and the emergence of immune-mediated diseases. Research has been done to clarify the processes by which helminth-derived molecules can inhibit immunological disorders. This study aimed to evaluate the ability of *Trichinella spiralis* chitinase (Ts-chit) to ameliorate the symptoms of allergic airway inflammation.

**Methods:**

Recombinant *Trichinella spiralis* chitinase (rTs-chit) was expressed in *Escherichia coli* BL21, and its structural homology to murine acidic mammalian chitinase (AMCase) was comprehensively analyzed. The expression of Ts-chit was examined across all *T. spiralis* life stages. To explore its immunomodulatory potential, a murine model of allergen-induced airway inflammation was established. The effects of rTs-chit were evaluated by assessing airway hyperresponsiveness and cytokine profiles in bronchoalveolar lavage fluid and performing detailed histopathological and immunohistochemical analyses.

**Results:**

Recombinant Ts-chit (rTs-chit) was successfully expressed in *E. coli* BL21, showing strong structural similarity to murine acidic mammalian chitinase (AMCase). Expression profiling revealed that Ts-chit is present throughout all stages of the *T. spiralis* life cycle. In an allergic airway inflammation model, rTs-chit reduced weight loss and lung inflammation, lowering inflammatory cell infiltration and Th2 cytokines (IL-4, IL-5, IL-13) while increasing the immunosuppressive cytokine IL-10. Additionally, rTs-chit treatment decreased the expression of GATA3, arginase-1, MCP-1, CCL-11, and AMCase, along with reducing OVA-specific IgE, IgG, and IgG1 levels, suggesting its potential as an immunomodulatory agent.

**Conclusions:**

This study highlights rTs-chit’s potential as a therapeutic agent for allergic airway diseases, leveraging its structural similarity to host chitinases to regulate Th2 responses and inflammatory pathways. The findings provide new insights into helminth-derived proteins as promising candidates for immune-based therapies.

**Graphical Abstract:**

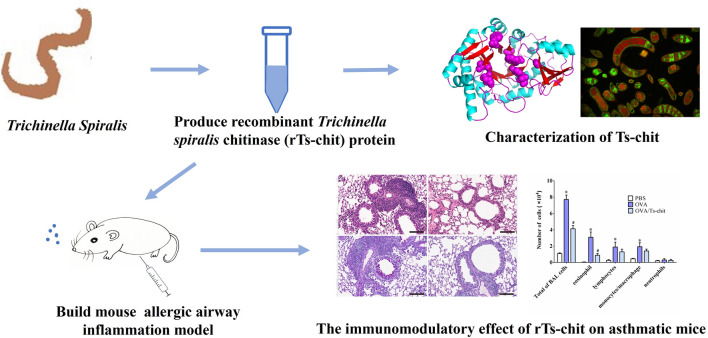

## Background

Currently, the rising incidence of allergic asthma has become a major hygiene burden on a global scale. A chronic inflammatory illness of the respiratory system, it is known to cause increased airway responsiveness, wheezing, dyspnea, chest tightness, coughing, and other potentially fatal symptoms [1]. Asthma affects around 300 million people worldwide, accounts for nearly a quarter of a million fatalities annually, and is expected to affect 400 million people by 2025 [2, 3].

Despite its long-term effectiveness in controlling asthma, beta-2 agonists and inhaled corticosteroids can cause some patients to become resistant to them. Additionally, the harmful side effects of steroids over the long term continue to deter patients from using them [4]. Future research on allergic asthma should therefore develop efficient targeted therapies based on the patient's condition.

Epidemiological investigation describes a reverse correlation between helminthic infection and allergic diseases including asthma, allergic rhinitis, and atopic dermatitis [5, 6]. A series of experimental studies supported the useful roles of helminths in alleviating the pathological changes of allergic diseases [7, 8]. Secretion with immunosuppressive effects in allergic diseases has been identified from different worms such as *Schistosoma japonicum*, *Heligmosomoides polygyrus*, *Trichuris suis*, and *Brugia malayi* [9–12].

A significant zoonotic parasitic nematode known as *Trichinella spiralis* is found all worldwide. It develops in a single host throughout its entire life cycle, unlike other parasites. A complex immune response is induced during the host invasion, and low doses of larvae infection can go undetected. However, the consumption of just a few hundred larvae can lead to serious clinical symptoms, posing a substantial risk to public health [13]. Current research on *T. spiralis* is primarily focused on creating an effective vaccine that will protect against infection or on utilizing its immune control characteristics to treat allergic or inflammatory illnesses in hosts [14, 15]. The pertinent investigations have shown that the excretion/secretion proteins (ESP) from *T. spiralis* and some of their constituent parts may be involved in allergic airway inflammation and have achieved some successes [16, 17].

Chitinases are enzymes that cleave chitin, a polysaccharide found in the cell walls of fungi and exoskeletons of arthropods, into low-molecular-weight chitooligomers. These enzymes are widespread in nature, produced by bacteria, fungi, plants, arthropods, and vertebrates [18, 19]. Chitinase plays a significant role in the immune response, particularly in the context of innate immunity [20]. It is mainly expressed by activated macrophages, highlighting its importance in immune defense mechanisms. Recent research has illuminated the immunomodulatory effects of chitinase, suggesting that it not only contributes to the direct degradation of chitin-coated pathogens but also influences the activation and polarization of macrophages and other immune cells, such as T helper cells and eosinophils [21–23]. Meanwhile, chitinases have also been identified in the parasites and play key role in the parasite’s life cycle. For example, chitinase has been reported in *Toxoplasma gondii* and involved in the cyst burden [24]. Another report also indicated that chitinases play a pivotal role in the transmission of malaria by facilitating the invasion of the mosquito midgut by the *Plasmodium* parasite [25]. The chitinases have also been linked to the pathogenesis of various human diseases, including bronchial asthma, chronic obstructive pulmonary disease (COPD), nonalcoholic fatty liver disease, and neurodegenerative disorders such as Alzheimer's disease and amyotrophic lateral sclerosis [23, 26–29]. Commonly, chitinase is thought to play a role by improperly inducing inflammation and disrupting tissue remodeling. For example, chitinases have been shown to be linked to asthma and parasitic responses. It could be upregulated during Th2 inflammation driven by IL-13, particularly in asthmatic tissues, which suggests that asthma might be a parasite-independent anti-parasite response [30]. Thus, investigating chitinase's immunomodulatory functions is vital for advancing our understanding of immune responses and developing targeted interventions, which could also shed light on its dual role in pathogen defense and its potential involvement in the pathogenesis of various inflammatory diseases.

Here, the chitinase domain-containing protein (Ts-chit, GenBank: KRY38634.1) derived from *T. spiralis* excretory-secretory proteins (ESP) was successfully cloned and expressed in the *Escherichia coli* BL21. The characteristics of Ts-chit were thoroughly investigated. Furthermore, the therapeutic potential of recombinant Ts-chit (rTs-chit) was evaluated for its ability to ameliorate allergic airway inflammation induced by alum-adjuvated ovalbumin (OVA). The results indicate that rTs-chit may have promising applications in the treatment of allergic airway inflammation, potentially paving the way for new therapeutic strategies targeting allergic conditions.

## Methods

### Animals

Eight-week-old female BALB/c mice were bought from Wushi Laboratory Animal Corporation and kept in a specified pathogen-free environment. The use of animals and experimental protocols in this study were approved by the Life Science Ethics Committee, Putian University (no. 2023 (008)).

### Phylogenetic analysis and three-dimensional structure of Ts-chit

The amino acid sequences of Ts-chit were compared with other chitinase from other *Trichinella* species, nematodes, human, and mouse deposited in the GenBank. All of the sequences were aligned using MEGA 7.0. The phylogenetic relationship between sequences was analyzed using maximum parsimony (MP). The SWISS-MODLE server was used to forecast and analyze the 3D structure models of Ts-chit. Pymol software was used to align Ts-chit and mus AMcase in three dimensions.

### Worm and protein preparation

*Trichinella spiralis* parasite (ISS534) used in this study was maintained by serial passages in BALB/c mice every 6 months. Muscle larvae (ML) were collected from the muscles of BALB/c mice using 0.9% pepsin and 1% HCl. The infective larvae (IIL) and adult worms (AW) were collected from the small intestines of BALB/c mice after 6 h, 3 and 6 days post-infection (dpi). The newborn larvae (NBL) were collected from 6dpi AW cultured medium as described [31]. The soluble worm extracts and ML excretion/secretion proteins were prepared by conventional methods [16, 31], and the concentration of these proteins was measured using the Bradford method.

### Production of recombinant Ts-chit and anti-rTs-chit serum

Using Trizol reagent, total RNA from *T. spiralis* worms was extracted, and the RNA was subsequently reverse-transcribed into cDNA. Using the *E. coli* system, the gene corresponding to the chosen Ts-chit was amplified and cloned into the vector pQE80L; 0.8 M isopropyl β-D-1-thiogalactopyranoside (IPTG) was used to stimulate the expression of rTs-chit for 5 h at 37 °C. Ni–NTA resin columns were used to separate the Ts-chit from the inclusion bodies proteins. Gradient dialysis was used to renature the pure protein for additional research. Following the analysis and identification of rTs-Chit proteins by SDS-PAGE and Western blotting, the quantities of the purified proteins were determined using the Bradford technique. Fifteen BALB/c mice were used to generate anti-rTs-chit serum. Each mouse received a subcutaneous injection of 20 µg of recombinant Ts-chit (rTs-chit) protein emulsified with an equal volume of complete Freund adjuvant for the initial immunization. For subsequent booster injections, administered on days 14 and 28, the mice were injected with 20 µg of rTs-chit emulsified with incomplete Freund adjuvant. All injections were performed at multiple subcutaneous sites. Blood samples were collected from the mice on day 42, and the sera were separated and stored at −20 °C until further analysis [32].

### Chitinase assay

In this study, Schales' reagent was employed to assess the chitinase activity of rTs-chit by reacting with N-acetylglucosamine (GlcNAc) released during chitin hydrolysis, resulting in a colorimetric change proportional to enzyme activity. The reagent was selected for its high sensitivity, reproducibility, and simplicity in quantifying GlcNAc, making it a reliable method for evaluating chitinase activity. The experimental procedure was as follows: 1% colloid chitin was incubated on a 96-well microtiter plate with escalating concentrations of chitinase solutions. After centrifugation at 4 °C, 100 μl of the supernatant was transferred to a new plate. Subsequently, 100 μl of Schales’ reagent, a mixture of 0.5 M sodium carbonate and 0.5 g/l potassium ferricyanide in water, was added. After 15 min of incubation at 100 °C and chilling, the plate was measured for absorbance at 420 nm. N-acetyl-D-glucosamine at 50 mM was utilized as a positive control.

### Western blot analysis

Western blot analysis was carried out in accordance with earlier research. SDS-PAGE was used to separate proteins (*T. spiralis* protein samples or mice lung samples), which were subsequently transferred onto nitrocellulose membranes (Millipore, USA). The membranes were then treated with the primary antibodies [1:100 dilutions of anti-rTs-chit serum, 1:1000 dilutions of anti-T-bet or anti-GATA-3 (Wanleibio, Shenyang, China) and GAPDH (Immunoway, USA)] at 37 °C for 1 h after being blocked with 5% skim milk. After washing with Tris-buffered saline with Tween 20 (TBST; 10–20 mM Tris, 150 mM NaCl, 0.05% Tween 20), the membranes were incubated with HRP-conjugated goat anti-mouse IgG (1:10000; Southern Biotech). The membranes were then treated with 3,3′-diaminobenzidine tetrahydrochloride (DAB; Sigma) or ultrasensitive ECL chemiluminescence reagent (Sangon Biotech, Shanghai, China) followed by washing with deionized water. Finally, the bands were visualized and quantified by an imager (JP-K300, Jiapeng technology, Shanghai, China) and analyzed with Image J 1.46r software.

### Analysis of Ts-chit gene transcription using RT-PCR

From *T. spiralis* in its various phases, including ML, IIL, AW, and NBL, total RNA was isolated. RT-PCR was used to examine the *Ts-chit* gene at each stage, as described throughout [33]. Amplifying *T. spiralis* GAPDH (GenBank accession no. AF452239) was used as the positive control.

### Immunofluorescent assay (IFA)

IFA was utilized to confirm the expression of Ts-chit at various *T. spiralis* stages. Paraformaldehyde was used to fix and embed *T. spiralis* ML and AW in paraffin. Using a microtome, 2-m-thick sections were created. The sections were treated with anti-rTs-chit serum (1:50 dilutions) at 37 °C for 1 h after blocking with 5% normal goat serum. Following a phosphate-buffered saline (PBS) wash, they were treated with FITC-labeled goat anti-mouse IgG (1:100 dilution, Santa Cruz, USA), the nucleus was stained with propidium iodide (PI), and they were subsequently observed under an Olympus fluorescent microscope (Japan) [32].

### Murine OVA-induced allergic airway disease

**Fig. 1:**
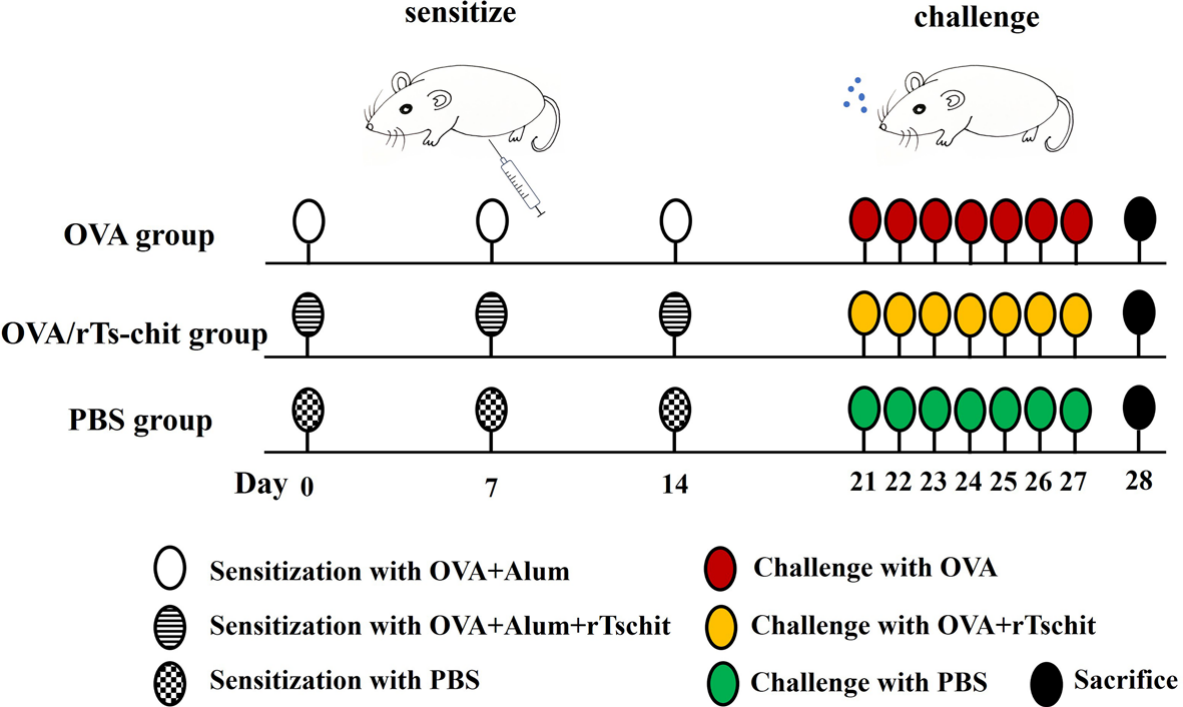
Experimental protocol for induction of allergic airway inflammation and treatment scheme

To evaluate the effects of Ts-chit on allergic airway disease in mice, ten female BALB/c mice were assigned to each of the three experimental groups. The OVA group was sensitized with 100 µg OVA (Sigma-Aldrich, Steinheim, Germany) formulated with 20 μg Alum Adjuvant (Thermo Fisher Scientific Inc., Shanghai, China) in a total volume of 200 μl by intraperitoneal injection (ip) on days 0, 7, and 14 and was challenged intranasally with OVA (100 μg in 50 μl) for 7 consecutive days (from day 21 to day 27). For the OVA/rTs-chit group, mice were intraperitoneally co-administrated 50 μg Ts-chit during the OVA sensitization on day 0, 7, and 14 and intranasally co-administrated 25 μg rTs-chit during the OVA challenge. In the PBS group, mice were stimulated using phosphate-buffered saline (PBS) at different times (Fig. 1). The use of BALB/c mice is supported by their well-established susceptibility to OVA-induced Th2-driven allergic inflammation, making them an ideal model for assessing the therapeutic potential of Ts-chit in modulating allergic airway disease.

### Evaluation of allergic airway symptoms and alterations in mouse weight

Allergic airway symptoms in each group of mice were noted and graded using the previously mentioned approach after the animals had been watched for 30 min following the last stimulation [17]. Zero points corresponded to no symptoms. One point was allocated to restlessness or scratching the nose. Open-mouth breathing and wheezing sounds scored two points. Three points were awarded for slow reply, severe respiratory difficulties, prostration, or unconsciousness. Mouse weight was recorded every 7 days until day 21 of the model-building process and then day 24 and day 28.

### Bronchoalveolar lavage

On the 28th day, mice were killed, and intratracheal bronchoalveolar lavage was performed by inflating the lungs with 500 µl sterile PBS. For each injection, the PBS remained in the lungs for 45 s, and then PBS was taken back to obtain the bronchoalveolar lavage fluids (BALFs). The BALFs were centrifuged at 3000 rpm for 10 min at 4 °C, and the supernatants were stored at −80 °C for cytokine detection. The total number of inflammatory BALF cells was determined by an automated cell counter (Bio-RAD, TC20^™^). For different cell counts, the cell pellets in BALF were stained using the Wright-Giemsa Stain Kit (Sigma-Aldrich, Shanghai, China).

### ELISA analysis of serum antibody and BALF cytokines in mice

Mouse serum was taken on the 28th day, and OVA-specific IgE was measured using mouse OVA sIgE ELISA kits (Wuhan Fine Biotech). The levels of OVA-specific IgG and OVA-specific IgG1 were also measured following earlier research [34]. The expression of IL-4, IL-5, IL-10, and IL-13 in BALF was analyzed following the manufacturer's protocol (Beyotime Biotechnology, Shanghai, China) and read at 450 nm on a microplate reader (Stat Fax^®^2100, Fisher Bioblock Scientific, France).

### Histology and immunohistochemistry (IHC) analysis

After lung lavage, the left lungs were fixed in 10% formaldehyde, embedded in paraffin, and sectioned for pathological analysis. The sections were then stained with the hematoxylin and eosin (H&E) and periodic acid shiff (PAS) reagent, according to standard protocols [35]. To examine the expression of GATA-3, T-bet, MCP-1, and arginase-1 in lung tissues, IHC analysis was also performed. The images of the stained sections were visualized using an optical biological microscope. The inflammation scores were evaluated, and the degree of infiltration was divided into 0 to 4 grades according to a previous study [17]. Briefly, score 0 represents no inflammatory cell infiltration, score 1 represents minor perivascular inflammation, score 2 represents moderate perivascular inflammation, score 3 represents increased perivascular and peribronchial inflammation and increased goblet cell hyperplasia, and score 4 represents severe perivascular, peribronchial, and interstitial inflammation with goblet cell hyperplasia in smaller and larger airways. Image-Pro Plus software was used to objectively examine the mucus distribution and IHC stained region.

### Real-time PCR for the quantification of chemokine expression

The real-time PCR was carried out according to the previous study with minor modifications. Briefly, total RNA was extracted from lung tissues using TRIzol^®^ reagent and then trascribed into cDNA. Real-time PCR was performed using the 7500 Fast Real-time PCR System, and results were calculated using the 2^−ΔΔCt^ method. Gene expression of arginase-1, MCP-1, CCL-11, and AMcase was described relative to the housekeeping gene GAPDH. The primer pairs used were as follows: arginase-1 (forward: 5’ CAGAAGAATGGAAGAGTCAG 3’, reverse: 5’ CAGATATGCAGGGAGTCACC3’); MCP-1 (forward: 5’ TTAAAAACCTGGATCGGAACCAA3’, reverse: 5’ GCATTAGCTTCAGATTTACGGGT3’); CCL-11 (forward:5’ CTGCTTGATTCCTTCTCTTTCCTAA3’, reverse: 5’GGAACTACATGAAGCCAAGTCCTT3’); AMcase (forward:5’ CCCTTGGCATATCCACTGA3’, reverse: 5’ACAGAATCCACTGCCTCCAG3’). Three independent assays were conducted, and each reaction was performed in triplicate.

### Statistical analysis

SPSS 27.0 was used to analyze the data, and the results are presented as the mean ± standard deviation (SD). One-way ANOVA was used to assess the differences between the various groups, with *P* < 0.05 considered statistically significant.

## Results

### Sequence alignment and phylogenetic analysis of Ts-chit

**Fig. 2:**
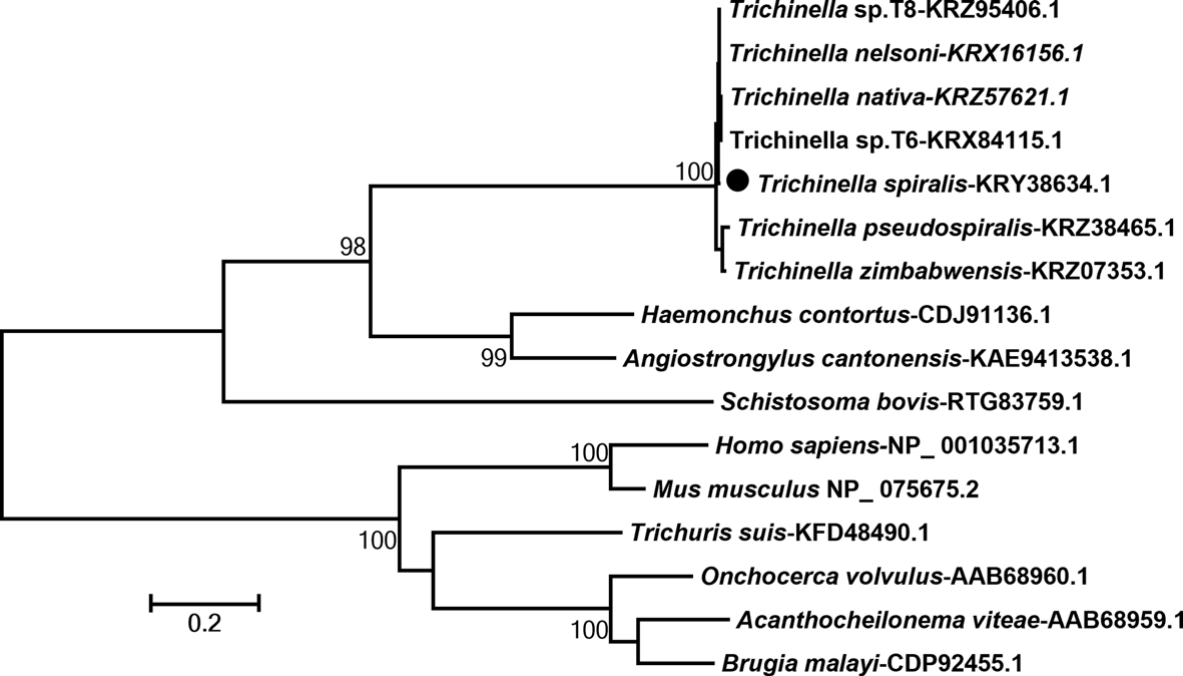
Phylogenetic tree of analysis of Ts-chit. Phylogenetic relationship of Ts-chit with chitinase of other nematodes, mouse, and human with maximum parsimony method and drawn with MEGA 7.0. Bootstrap values > 60 are indicated on branches. The sequence indicated with a solid circle was Ts-chit protein (KRY38634.1) cloned in this study

**Fig. 3:**
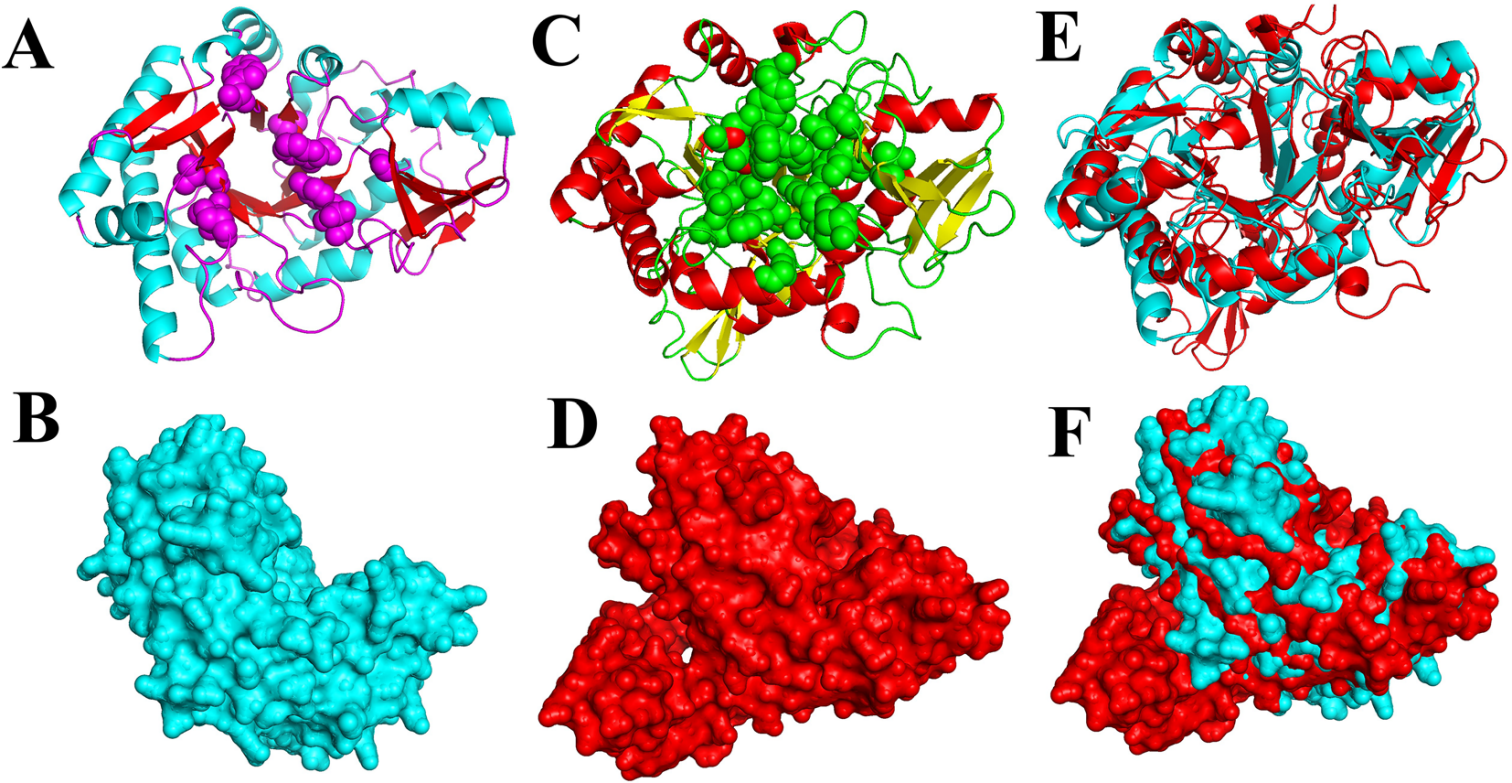
Three-dimensional structure of Ts-chit. Overall cartoon structure of Ts-chit (**A**) (α-helices in blue, β-strands in red, active sites in purple spheres). Surface structure of Ts-chit (**B**). Overall cartoon structure of mus AMcase (**C**) (α-helices in red, β-strands in yellow, active sites in green spheres). Surface structure of Ts-chit (**D**). Alignment of Ts-chit and Mus AMcase in cartoon structure (**E**) and surface structure (**F**)

The phylogenetic analysis result is shown in Fig. 2. All Ts-chit from *Trichinella* was grouped in one clade, which confirmed that the Ts-chit (KRY38634.1) from *T. spirals* is a typical chitinase. To determine whether Ts-chit has any characteristics that could make it a molecular mimic of host chitinase when it functions as an antigen, we compared the three-dimensional structure of Ts-chit with that of mouse chitinase (AMCase). The findings showed that they have some structural and enzymatic activity pocket similarities (Fig. 3).

### Expression and purification of rTs-chit protein

**Fig. 4:**
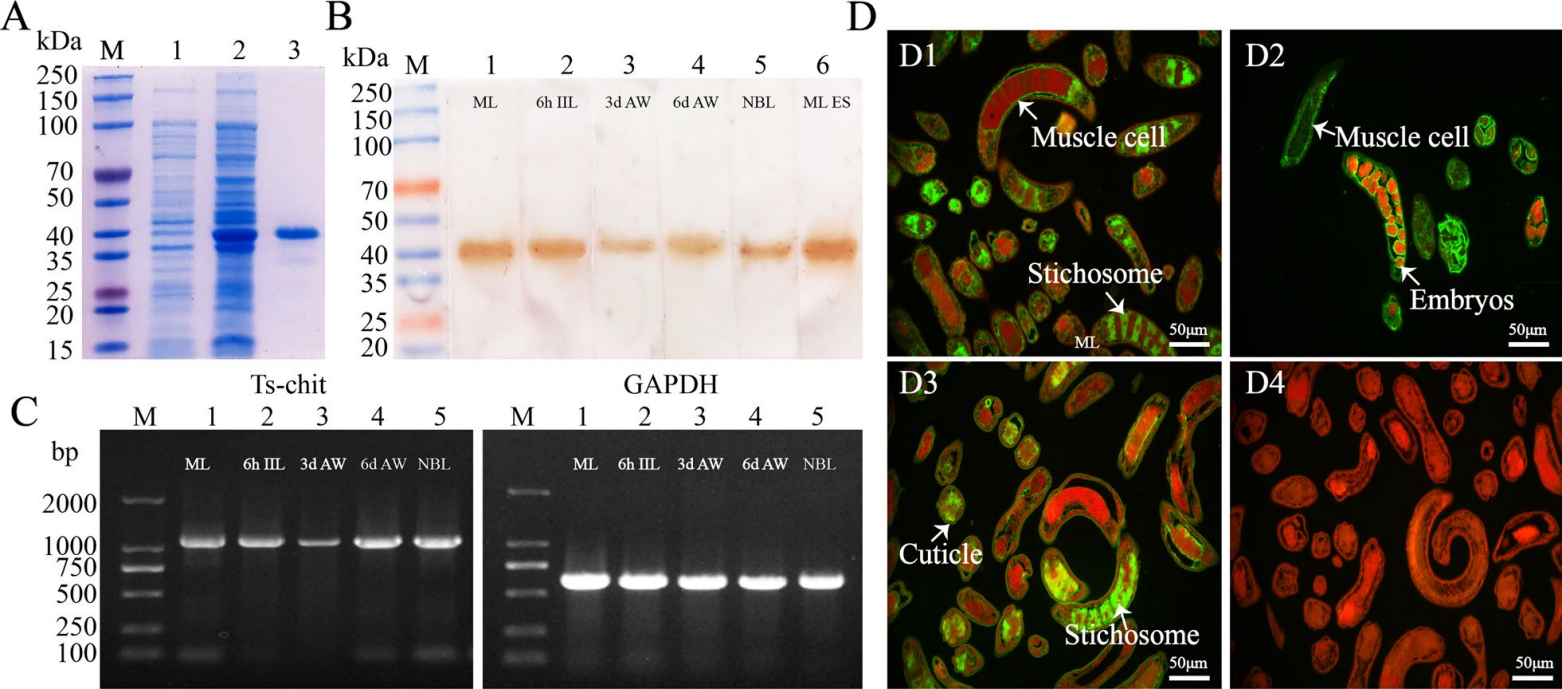
Identification of rTs-chit protein and expression of Ts-chit in different stages. **A** SDS-PAGE analysis of rTs-chit: Lane M, protein molecular weight marker; Lane 1, lysate of uninduced *Escherichia coli* BL21 carrying pQE-80L/Ts-chit; Lane 2, lysate of IPTG-induced *E. coli* BL21 carrying pQE-80L/Ts-chit; Lane 3, purified rTs-chit protein (~ 41 kDa). **B** Western blot analysis of native Ts-chit. **C** RT-PCR analysis of *Ts-chit* transcription (1092 bp). **D** Immunofluorescence localization of Ts-chit: D-1 and D-2, green immunostaining in ML and AW using anti-rTs-chit serum; D-3, positive control with infection serum showing staining in ML; D-4, negative control with normal serum showing no staining. Nuclei are stained red. Staining intensity was assessed qualitatively by comparison with the negative control, with stronger staining indicating higher expression of Ts-chit. Scale bars: 50 μm. (magnification 400 ×)

The fragment (1092 bp) of *Ts-chit* was obtained and cloned into the pQE-80L expression vector using a seamless clone kit. After induction by IPTG (Fig. 4A, Lane 1), rTs-chit fusion protein was obtained from the *E. coli* BL21 harboring pQE-80L/Ts-chit. The recombinant protein was then purified using the Ni–NTA-Sefinose Column, and a single band was observed on SDS-PAGE analysis (Fig. 4A Lane 3). The molecular weight of rTs-chit protein is about 41 kDa, congruent with its predicted size.

### rTs-chits devoid of enzymatic activity are expressed in various phases of *T. spiralis*

The renatured rTs-chit did not exhibit any enzymatic activity, according to Schales' chitinase detection technique (data not provided). That might be because Ts-chit does not have the traditional pattern structure, DXDXE. Western blot was carried out to investigate the expression of native Ts-chit in different *T. spiralis* stages. Results showed that rTs-chit protein was recognized by anti-rTs-chit serum in crude proteins of all *T. spiralis* life cycle stages and in the ES proteins (Fig. 4B). The Ts-chit mRNA transcript was also detected at all *T. spiralis* life cycle phases (Fig. 3C). The immunofluorescence assay revealed strong green immunostaining in the muscle cells and stichosome of the ML section (Fig. 4D1) as well as in the muscle cells and embryos of the AW section (Fig. 4D2). While the nematode sections were probed with the *T. spiralis* infection serum, immunostaining was also detected in the cuticle and stichosome of ML sections (Fig. 4D3), and no intense staining was observed on the ML section when probed with the normal mouse serum (Fig. 4D4). These results reveal that different developmental stages of *T. spiralis* express Ts-chit, which could indicate the importance of this molecule for the development of all *T. spiralis* life cycle stages.

### rTs-chit relieved allergic airway symptoms in mice

**Fig. 5:**
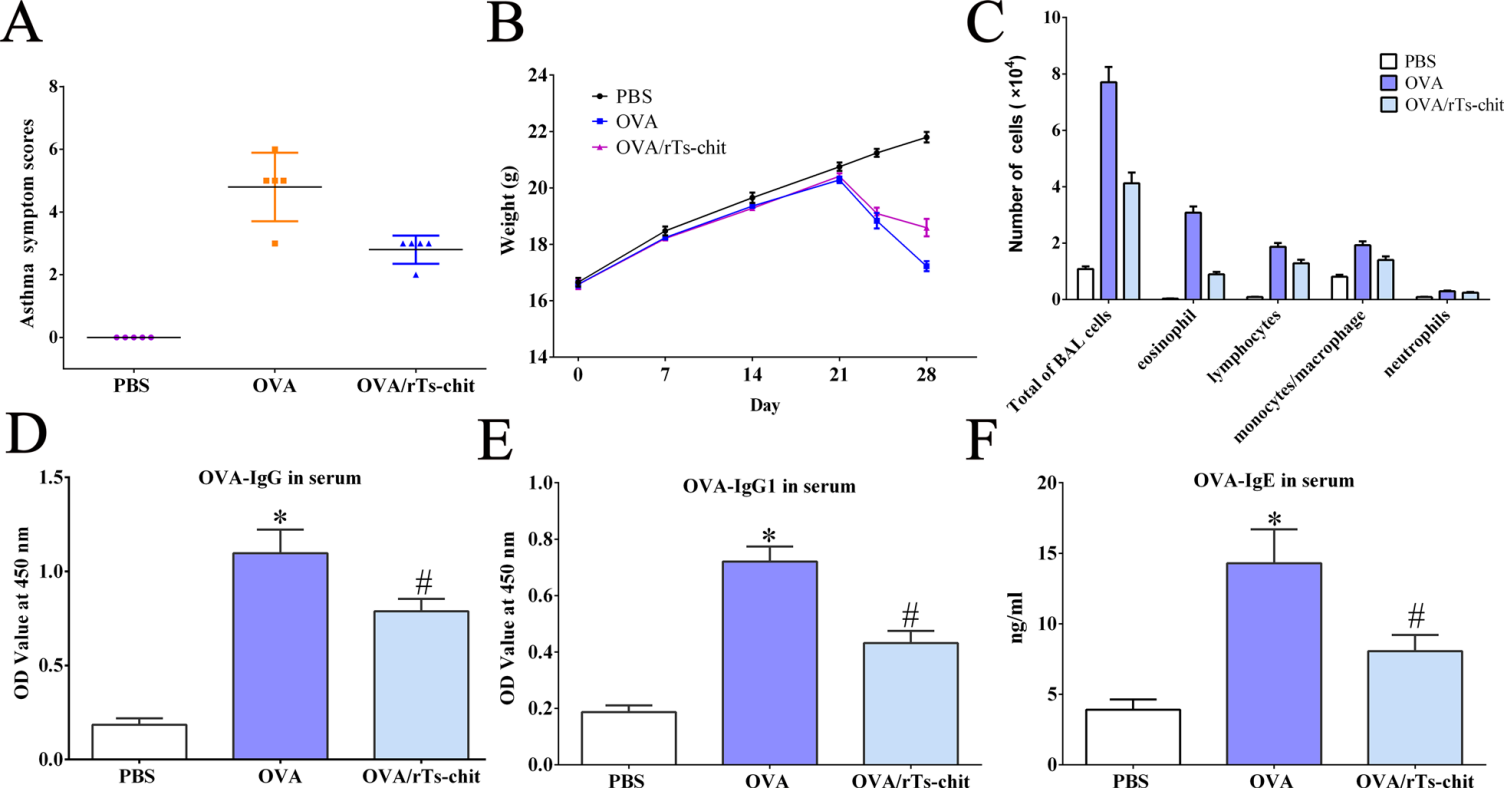
Symptom score (**A**) at day 28 and weight (**B**) during the OVA-induced allergic airway model, inflammatory cell count in BALF (**C**), and serum levels of OVA-IgG (**D**), OVA-IgG1 (**E**), and OVA-IgE (**F**). Data are mean ± SD (*n* = 10 per group). *P < 0.05 compared with the PBS group, #*P* < 0.05 compared with the OVA group

The allergic airway symptoms of mice were recorded and scored after the last stimulation by observing them for 30 min. First, no symptoms were observed in the PBS group, and the score was 0. In the OVA group, the symptoms of sneezing, shortness of breath, scratching their noses, and prostration were observed in the mice. After calculation, the symptom score of the OVA group was significantly higher than that of the PBS group (4.8 vs 0, *P* < 0.05). Meanwhile, the score of OVA/rTs-chit group was significantly lower than that of the OVA group (2.8 vs 4.8, *P* < 0.05) (Fig. 5A). The results suggested that an allergic airway inflammation model had been established, and the administration of rTs-chit could relieve allergic airway symptoms. The weight of mice in each group was also recorded. In the PBS group, body weight increased steadily throughout the experiment, reflecting the normal growth trajectory of BALB/c mice under standard laboratory conditions. In contrast, after day 21, weight loss was observed in both the OVA and OVA/rTs-chit groups. However, the extent of weight loss was significantly greater in the OVA group than in the OVA/rTs-chit group, indicating that rTs-chit treatment effectively alleviated OVA-induced weight reduction (Fig. 5B).

The inflammatory cells collected from BALF were also counted, and the results further confirmed that rTs-chit could relieve the lung inflammation in the allergic airway mice (Fig. 5C). They showed that eosinophils, macrophages, lymphocytes, and neutrophils were significantly higher in the OVA group than in the PBS group (*P* < 0.05). Moreover, the number of inflammatory cells in the OVA/rTs-chit group were significantly less than in OVA group (*P* < 0.05).

The serum levels of OVA-specific IgE, IgG, and IgG1 were measured, showing the highest expression in the OVA group, followed by the OVA/rTs-chit group, with the lowest levels observed in the PBS group. Significant differences were found between all groups (*P* < 0.05), suggesting that OVA treatment exacerbated the allergic airway response, while rTs-chit administration alleviated it.

### Histopathology of the lungs

**Fig. 6:**
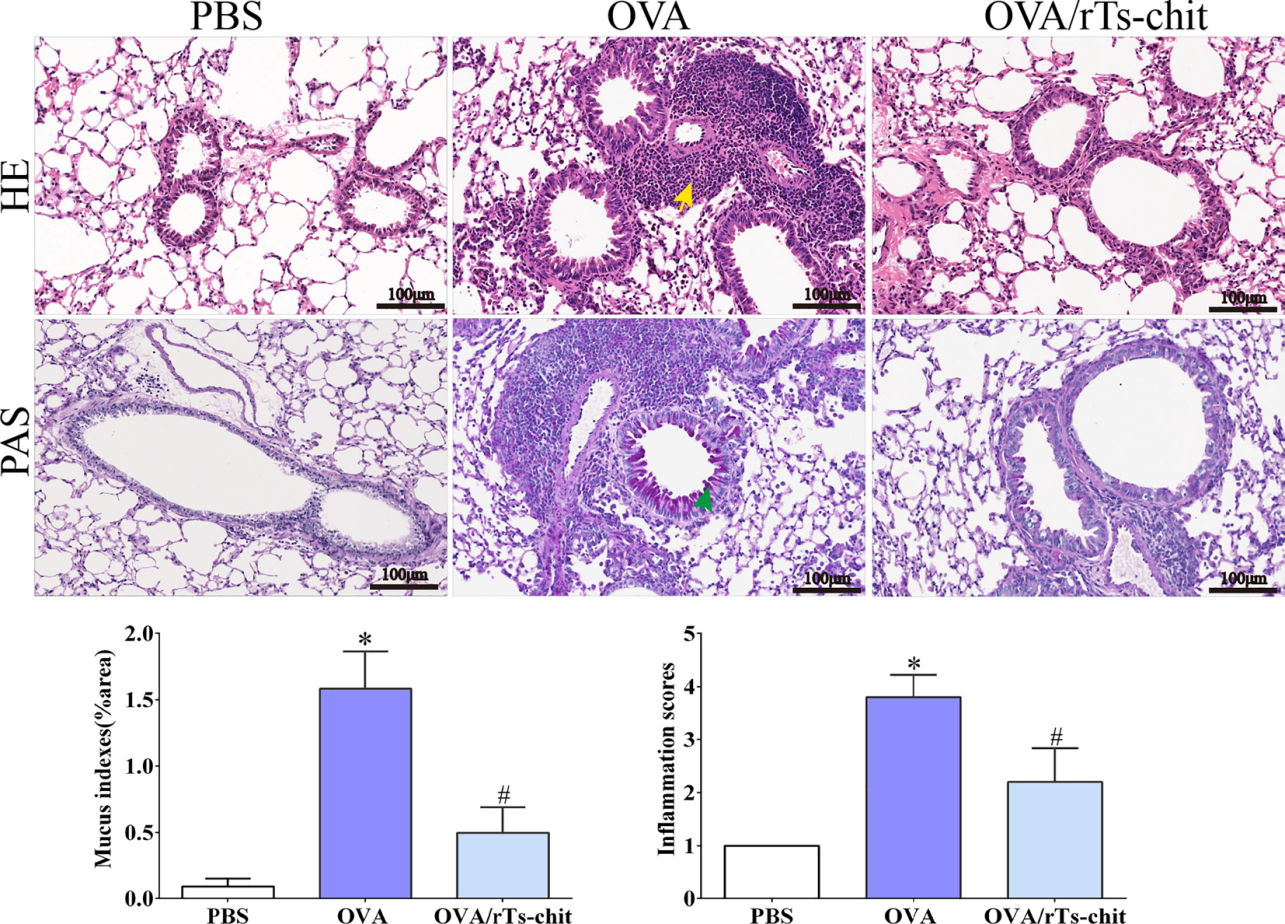
Histological changes of lungs. Lung tissue section of PBS, OVA, and OVA/rTs-chit group was stained with hematoxylin and eosin (H&E) or periodic acid shiff (PAS). Perivascular and peribronchial infiltration was scored. The yellow arrow represents inflammatory infiltrating cells, and the green arrow represents goblet cells. *P < 0.05 significant difference compared with the PBS group, #*P* < 0.05 compared with the OVA group. Scale bars: 100 μm (magnification 200 ×)

Histological analysis (H&E staining and PAS staining) (Fig. 6) showed that the alveolar structures in the mice were clearly visible. There was no inflammatory cell infiltration or goblet cell proliferation in the control group. Meanwhile, many inflammatory cells, goblet cells, and mucus were detected in the OVA group. Statistical analysis showed that the lung inflammation in the OVA group was significantly higher than that in the OVA/rTs-chit group and PBS group (*P* < 0.05, *P* < 0.05).

### Ts-chit attenuated Th2 responses in lung tissues

**Fig. 7:**
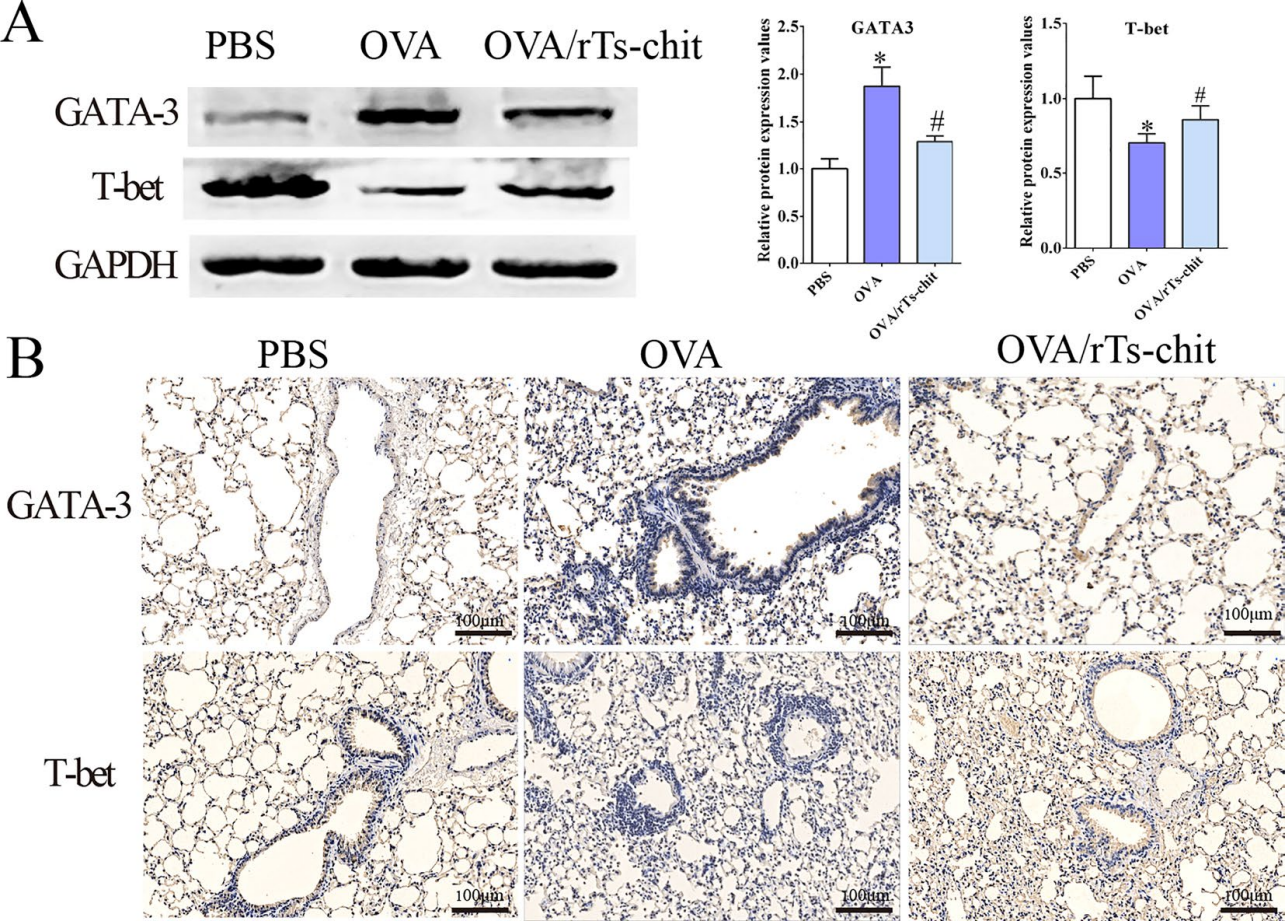
Expression of T-bet and GATA-3 in the lung. Western blotting was used to analyze the T-bet and GATA-3 in the lung, GAPDH as the control (**A**). IHC was used to analyze the T-bet and GATA-3 (**B**). *P < 0.05 significant difference compared with the PBS group. ^#^*P* < 0.05 compared with the OVA group

**Fig. 8:**
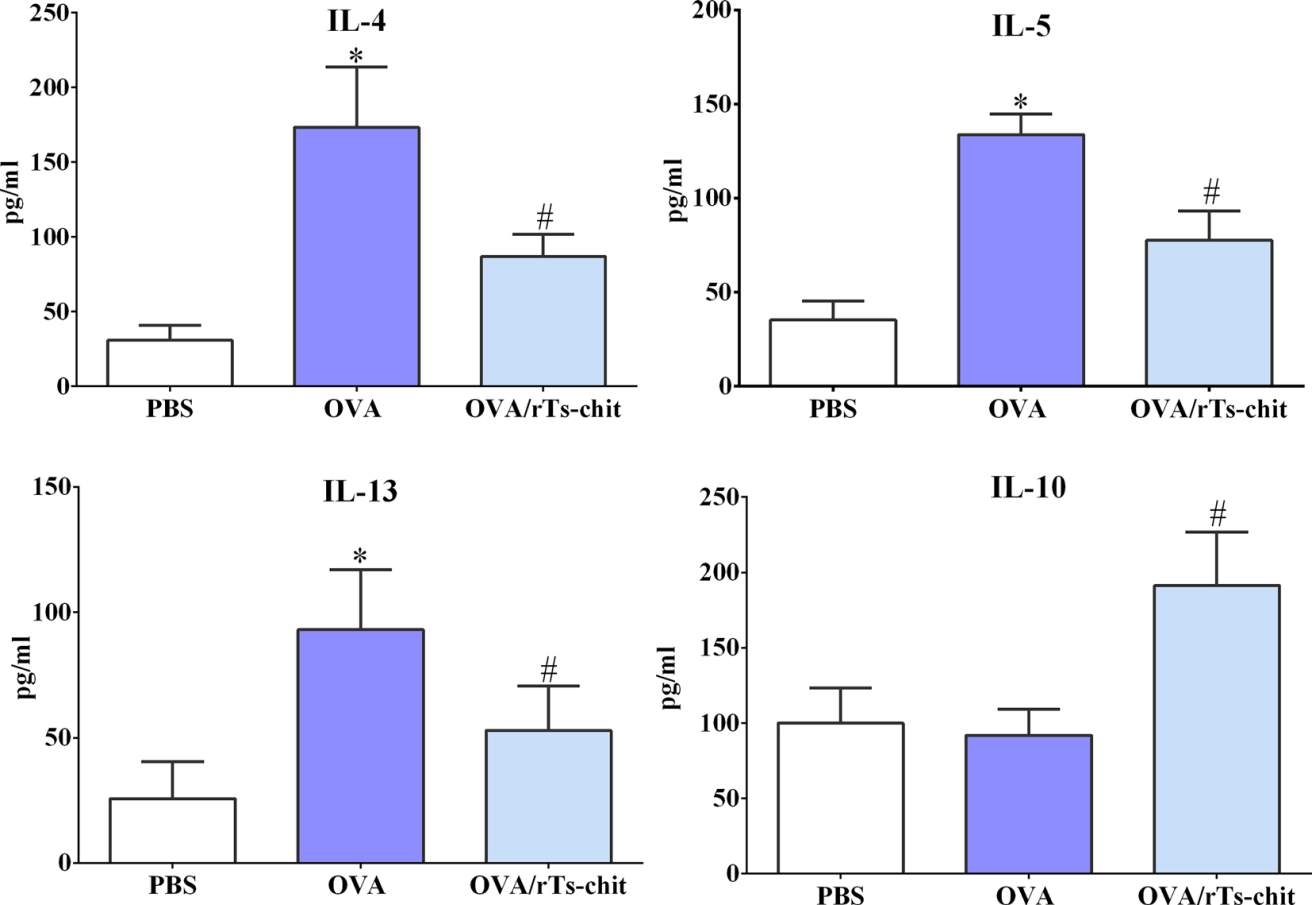
Cytokine levels in bronchoalveolar lavage fluid (BALF). Data are expressed as mean ± SD. *P < 0.05 significant difference compared with the PBS group. #*P* < 0.05 compared with the OVA group

The levels of Th1- and Th2-specific factors (T-bet and GATA-3) in the lung were identified using Western blotting and IHC to investigate the intervention effects of rTs-chit on the Th1/Th2 immune response in the allergic airway mice. When comparing the OVA group to the PBS group, there was a clear rise in GATA3 expression in both essays (*P* < 0.05), whereas a substantial decrease in T-bet expression was observed in the OVA group. rTs-chit administration may increase T-bet expression while decreasing GATA3 expression compared to the control group (Fig. 7). ELISA was used to identify the common cytokines of Treg cells (IL-10) and Th2 cells (IL-4, IL-5, and IL-13) in BALFs. Th2 cell cytokines were notably greater in the OVA group than in the PBS group. Compared to the OVA group, the OVA/rTs-chit groups showed significantly lower levels of IL-4, IL-5, and IL-13 (*P* < 0.05) and a noticeable rise in IL-10 (Fig. 8). These findings suggested that by lowering the Th2 immune response in the lungs, rTs-chit treatment could lessen the symptoms of allergic airway inflammation.

### rTs-chit interferes with chemokines and murine chitinase in allergic airway model

**Fig. 9:**
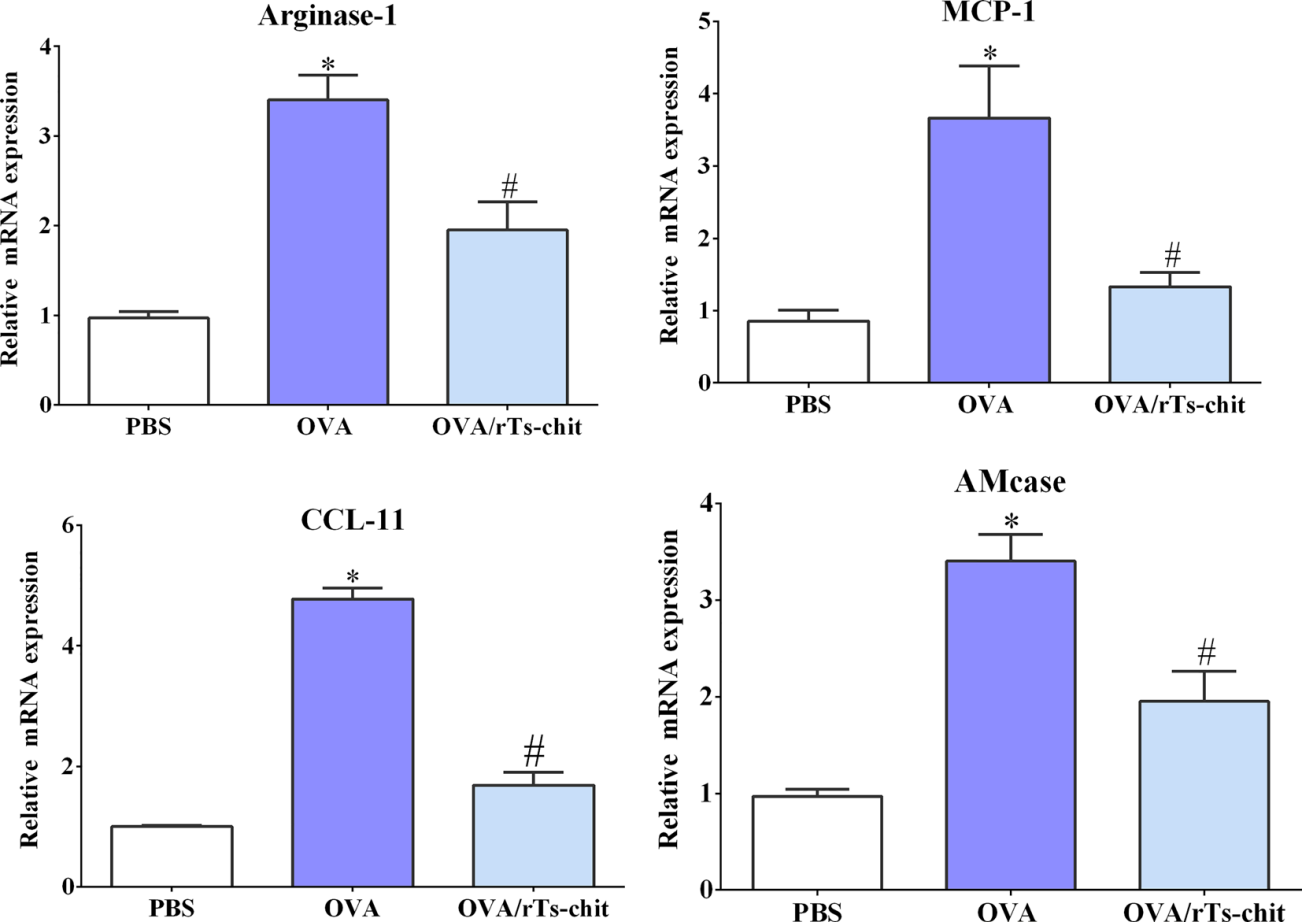
Relative mRNA expression of arginase-1, CCL-2 (MCP-1), CCL-11 (eotaxin-1), and AMcase in the lung tissue. Data are expressed as mean ± SD from three separate experiments. *P < 0.05 indicates a significant difference compared with the PBS group. ^#^*P* < 0.05 compared with the OVA group

**Fig. 10:**
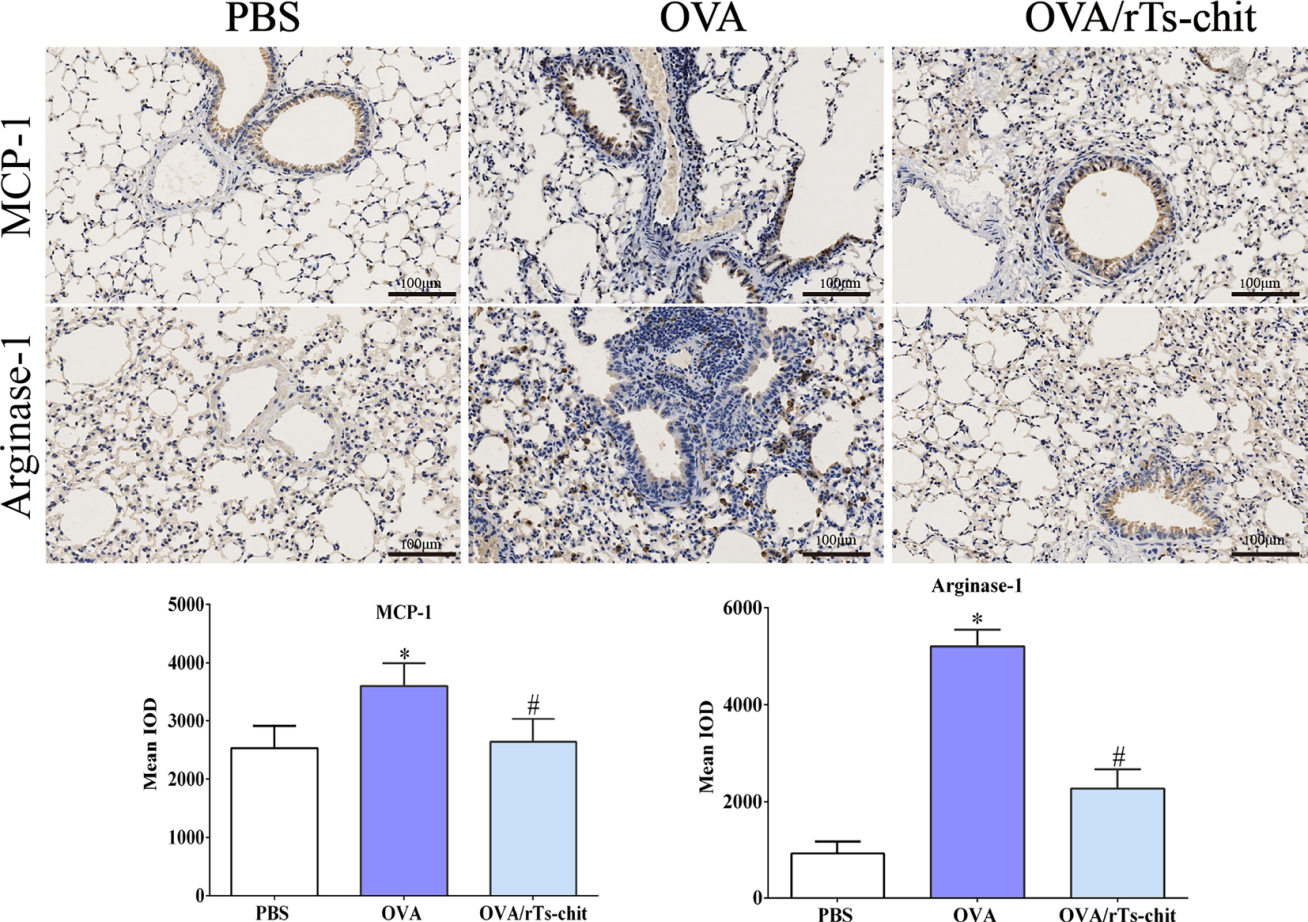
IHC analyzed the expression of MCP-1 and arginase-1 in the lung. The intensity and distribution of staining were quantitatively assessed using Image-Pro Plus software to provide an objective analysis of the immunoreactivity. Scale bars: 100 μm (magnification 200 ×), *P < 0.05 indicates a significant difference compared with the PBS group. ^#^*P* < 0.05 compared with the OVA group

Real-time PCR analyses were carried out utilizing mRNA obtained from lung samples of three groups to evaluate the expression levels of the CCL11 (Eotaxin-1), CCL2 (MCP-1), arginase-1, and AMCase transcripts. Compared to the mRNA expression levels in PBS mice, OVA-sensitization significantly increased the mRNA expression levels of eotaxin, MCP-1, arginase-1, and AMcase in the lungs (Fig. 9). rTs-chit treatment, however, significantly reduced the transcript levels of these genes in contrast to the expression seen in the OVA-sensitized group. IHC was used to further explore the expression position of MCP-1 and arginase-1 in the lung. The expression trends of MCP-1 and arginase-1 agreed with the real-time PCR results (Fig. 10).

## Discussion

To maintain their existence, molecules produced by parasites employ a variety of strategies to regulate the host immune responses [36, 37]. These mechanisms include limiting the development of allergic disorders and preventing excessive inflammatory reactions. A notable strategy involves the upregulation of immunosuppressive cytokines such as IL-10, which plays a crucial role in dampening inflammation and promoting immune tolerance. Studies have shown that helminths can induce IL-10 to suppress Th2-driven airway inflammation, which aligns with their ability to persist within hosts while mitigating excessive immune responses. Given these properties, parasite-derived molecules may offer safer and more effective therapeutic options for managing allergic diseases than conventional chemical treatments.

An increasing number of studies suggest that *T. spiralis*, a tissue-dwelling nematode with a global distribution, and its ES antigens function as inflammatory modulators to help against autoimmune and allergy disorders [14, 16, 17]. In this study, we focused o identifying and characterizing chitinase protein secreted by *T. spiralis* (Ts-chit). Ts-chit is expressed at multiple stages (ML, IIL, AW NBL, and ES) in *T. spiralis*, indicating its essential role in the parasite's development.

Chitinases are glycosyl hydrolases (GH) capable of degrading chitin polymers to low-molecular-weight chitooligomers [38, 39]. *Trichinella spiralis* chitinase is classified as belonging to GH family 18 based on similarity of amino acid sequences. It is distinguished by an enzymatic core consisting of eight β-strands that form a barrel, encircled by α-helices. The conserved DxDxE sequence, which normally forms the active site, is absent from it. Its role as an enzyme is called into question by this absence. Interestingly, it has been demonstrated that certain dormant chitinases, including chitinase 3-like protein 1 (commonly referred to as CHI3L1 or YKL-40), can regulate inflammation without the need for enzymatic activity. Particularly in cases of asthma and COPD, CHI3L1 is associated with the resolution of inflammation, tissue remodeling, and immune response control. Its presence in airway epithelium correlates with reduced eosinophilic inflammation and improved lung function, demonstrating the significant role non-active chitinases can play in managing inflammatory diseases [40, 41]. Remarkably, research on *Trichuris suis* chitinase shows that it maintains immunomodulatory characteristics even after heat inactivation, suggesting that chitinase can have effects apart from its enzymatic activity. Furthermore, this research underscores the importance of structural mimicry in immune modulation. High-resolution x-ray crystallography revealed that the three-dimensional structure of *T. suis* chitinase closely resembles that of AMCase. This structural similarity likely allows the helminth-derived chitinase to modulate host chitinase activity and suppress airway inflammation, potentially through mechanisms such as competitive interference or functional modulation of host enzymatic pathways [42]. Similarly, our results imply that rTs-chit, while not enzymatically active, may utilize similar structural mimicry mechanisms to modulate immune responses. This structural mimicry could enable rTs-chit to interact with host receptors or interfere with host chitinase functions, thereby contributing to its observed anti-inflammatory effects. However, the lack of enzymatic activity in rTs-chit restricts fully understanding its immunomodulatory mechanisms. Future studies should focus on engineered Ts-chit variants with restored enzymatic function to evaluate whether it boosts or alters immunoregulatory effects. This helps understand the interplay of structural mimicry and enzymatic activity, advancing parasite-derived molecules' therapeutic use. Also, further research is needed to clarify the molecular mechanisms of *T. spiralis* chitinase's immunomodulation. It is unclear whether host chitinase mimicry alone causes these effects or whether other mechanisms like receptor binding or downstream signaling modulation are involved. The structural resemblance between *T. spiralis* and host chitinases offers a good basis for better understanding its immunoregulatory potential.

In the current investigation, mice exhibited evident symptoms of allergic airway inflammation and weight loss following OVA induction. After rTs-chit intervention, the mice demonstrated alleviated symptoms and an increase in body weight, suggesting that rTs-chit possesses potential for ameliorating allergic airway inflammation. The primary pathological alteration symptoms of allergic airway inflammation include goblet cell hyperplasia and mucus hypersecretion in the airway, alongside the infiltration and proliferation of inflammatory cells [17]. Our findings demonstrated that the allergic airway inflammation mice underwent these common pathological alterations. rTs-chit antigens efficiently lowered goblet cell aberrant growth and mucus secretion as well as the recruitment and proliferation of inflammatory cells in the lung.

Allergic airway inflammation is traditionally treated as a Th2 illness associated with increased IgE and eosinophilic inflammation, which is essential to the pathogenesis of asthma in the airway [43]. Th2 cytokines—including IL-4, IL-5, and IL-13—are strongly linked to mucus production and airway inflammation, playing vital roles in the development and progression of allergic asthma [44]. As a common Th2-inducing cytokine, IL-4 is essential for B cells to flip their IgE isotype [45]. IL-5 has a crucial role in the differentiation, maturity, and survival of eosinophils, which leads to an excess of IgE and mucus. It is also intimately linked to airway eosinophilia and hyper-reactivity [46]. IL-13 is a major cause of airway inflammation and is linked to goblet cell hyperplasia and mucus production in airway epithelial cells [45, 47]. Research indicates that anti-cytokine treatments inhibiting Th2-related cytokines can have substantial clinical benefits, suggesting that a reduction in Th2 cytokine output may provide therapeutic advantages for patients with allergic airway inflammation.

Multifunctional immunosuppressive cytokine IL-10 is essential for controlling immune responses. It modulates the inflammatory response by inhibiting pro-inflammatory cytokines such TNF-α, IFN-γ, and IL-6 [48]. Crucially, IL-10 affects the growth and specialization of distinct immune cell types, such as T lymphocytes, B lymphocytes, and myeloid cells. The innate immune response depends heavily on myeloid cells, such as dendritic and macrophage cells [49, 50]. In addition to encouraging naïve T cells to differentiate into regulatory T cells (Tregs), which are necessary for immunological tolerance maintenance and autoimmunity prevention, IL-10 also improves myeloid cells' capacity to take on an anti-inflammatory phenotype [51]. For instance, IL-10 skews macrophages towards an M2 phenotype, which is associated with tissue repair and anti-inflammatory responses, and inhibits their production of pro-inflammatory mediators [51, 52]. To facilitate immunoglobulin production and class switching, IL-10 stimulates B cell proliferation, blocks apoptosis, improves differentiation, and upregulates MHC II expression [53]. Recent studies highlight the role of IL-10-producing regulatory B cells (Bregs) in allergic diseases [54, 55]. These IL-10 + Bregs promote the expansion of IL-10 + Tregs and help maintain the FoxP3 + Treg population, contributing to parasite-induced immunosuppression in allergic airway inflammation [54]. Successful allergen-specific immunotherapy has been linked with the induction of IL-10 [56], and several helminth antigens are known to induce of IL-10 production [57–59] both in vitro and in vivo.

During the challenge phase of allergy, lung macrophages upregulate arginase-1 expression, inducing an immunosuppressive response to limit the lung DC function and antigen-specific antibody production [60, 61]. We found a significant decrease in argninase-1 in the OVA/rTs-chit group compared to the OVA group. In the present study, compared to the OVA-challenged allergic airway inflammation group, mice treated with rTs-chit showed decreased Th2 cytokine expression, indicated by lower levels of IL-4, IL-5, and IL-13 in BALF, GATA-3 in lung tissue, and IgG1 in the serum. Furthermore, rTs-chit dramatically raised IL-10 expression in BALF. Our observations suggest that rTs-chit, through upregulating immunosuppressive cytokine IL-10 and downregulating Th2 cytokines, can effectively attenuate the allergic airway inflammation response.

Due to the toxic chemicals in their granules, eosinophils and neutrophils have been shown to directly damage lung tissue, contributing to the development and severity of allergic airway inflammation [62–64]. Thus, regarding the recruitment and accumulation of eosinophils and neutrophils in the airway experiencing an allergic reaction, chemokines released by airway epithelial cells and dendritic cells upon allergen exposure are crucial to the immune response [65, 66]. Our study findings show that rTs-chit can reduce airway eosinophil and neutrophil counts in OVA-sensitized and challenged mice, correlating with decreases in the levels of chemotactic factors such as eotaxin-1, MCP-1, and IL-5.

Acidic mammalian chitinase (AMCase), which is expressed in mice, plays a key role in mediating the Th2-driven inflammatory responses commonly associated with allergic airway inflammation and has been investigated as an indicator of disease severity. AMCase is secreted by macrophages and epithelial cells of the lung and gut. In the lungs, it is constitutively expressed and secreted into the airway lumen. IL-13 induces AMCase expression, and elevated AMCase mRNA and protein levels have been detected in BAL of OVA-induced asthmatic mice [67, 68]. Blocking AMCase activity by RNA interference suppresses ovalbumin-sensitized allergic asthma [69]. Anti-AMCase sera diminished the ability of IL-13 to stimulate eotaxin and monocyte-chemotactic protein1 (MCP-1). Friederike and coworkers demonstrated that *T. suis*-derived chitinase reduced the expression of AMCase and displayed immunomodulatory properties in inflammatory lung disease [42].

As evidence, we showed that rTs-chit treatment during sensitization decreased AMcase transcript levels in mice. Additionally, structural alignment analyses reveal similarities between *T. spiralis* chitinase and AMCase. Such structural mimicry suggests that rTs-chit may interfere with AMCase expression or activity, potentially through competitive inhibition or modulation of its functions within the inflammatory milieu of the lungs [42]. This interaction may alter the balance of inflammatory mediators, subsequently influencing the overall immune response in the context of allergic airway inflammation. Moreover, it has been demonstrated that rTs-chit downregulates Th2 cytokines, such as IL-13, and upregulates the immunosuppressive cytokine IL-10. This modulation may lead to a decreased demand for AMCase activity, as IL-13 is a critical driver of AMCase expression and is associated with the Th2-mediated inflammatory response observed in asthma [67]. By downregulating Th2 cytokines, rTs-chit may reduce the inflammatory stimuli that normally increase AMCase synthesis, resulting in a decrease in AMCase levels. Consequently, it remains to be determined whether the observed reduction in AMCase levels is a direct result of rTs-chit interference or a secondary effect stemming from an overall decrease in lung inflammation. Future studies are needed to disentangle these mechanisms.

In conclusion, we identified and characterized a chitinase protein of *T. spiralis* (Ts-chit) and its capacity to immunomodulate allergic airway inflammation in mice by upregulating the immunosuppressive cytokine IL-10 and downregulating Th2 cytokines. Lacking enzymatic activity, rTs-chit likely exerts its effects through non-enzymatic mechanisms such as structural mimicry or direct immune modulation. The structural similarity between Ts-chit and host chitinases may interfere with endogenous chitinase activity, contributing to its therapeutic effects. Compared to conventional treatments for airway inflammation, rTs-chit offers a unique mechanism of action, emphasizing its potential as a novel therapeutic strategy. Nevertheless, further studies are needed to unravel the intricate immune mechanisms underlying helminthic protein-mediated anti-inflammatory responses.

## Data Availability

No datasets were generated or analysed during the current study.
